# Mechanical Stress Reveals Asymmetry of Sodiation and Desodiation of Hard Carbon

**DOI:** 10.1002/cssc.202501272

**Published:** 2025-08-19

**Authors:** Stefan Mück, Dominik Kramer, Krishnaveni Palanisamy, Christine Kranz, Reiner Mönig

**Affiliations:** ^1^ Institute for Applied Materials Karlsruhe Institute of Technology Hermann‐von‐Helmholtz‐Platz 1 76344 Eggenstein‐Leopoldshafen Germany; ^2^ Institute of Analytical and Bioanalytical Chemistry Ulm University Albert‐Einstein‐Allee 11 89081 Ulm Germany

**Keywords:** hard carbon anodes, mechanical stress, Na^+^ storage mechanisms, pore filling, sodium‐ion batteries

## Abstract

Sodium‐ion batteries (SIBs) are an important and environmentally friendly drop‐in technology for widespread lithium‐ion batteries. Currently, hard carbon (HC) composite electrodes are considered to be the most promising anodes for commercial SIBs. Still, research is required to improve hard carbons to reduce irreversible losses during the first cycle and to gain a better understanding of how their structure correlates with their electrochemical performance. Herein, a HC composite electrode is investigated by *operando* substrate curvature measurements to determine mechanical stresses and infer volume changes during operation. These mechanical data in conjunction with electrochemistry are used to compare different reaction pathways proposed in the literature. It is observed that sodium loss in the first cycle does not lead to large changes in volume. The mechanical data agree with the model based on intercalation and pore filling. A strong asymmetry in the volume of the HC between sodiation and desodation is identified and explained by modifications of the model: intercalation and pore filling are mostly sequential, but both mechanisms interact so that sodium deposition into and dissolution from pores occur at different levels of intercalation.

## Graphical abstract

*Operando* substrate curvature measurements are used to monitor the mechanical stress of hard carbon composite electrodes during sodiation and desodiation. The observed asymmetry of the electrode volume between sodiation and desodiation is explained by an interaction between sodium intercalation and pore filling: during pore filling, the sodium concentration in the transport paths is higher than during pore depletion.

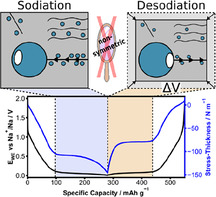

## 1 Introduction

For many applications, sodium‐ion batteries (SIBs) are considered to be promising alternatives to lithium‐ion batteries (LIBs). SIBs are of interest as a cost‐efficient drop‐in technology because of their improved sustainability compared to LIBs resulting from their nontoxic and abundant raw materials. The common anode material of LIBs is graphite. With sodium as the insertion ion, graphite only shows minor capacities due to the thermodynamic instability of sodium‐rich binary graphite intercalation compounds.^[^ ^1^ ^]^ For SIBs, hard carbon (HC) is the material of choice due to its low electrochemical potential (close to Na^+^/Na), high storage capacity (200–400 mAh g^−1^),^[^ ^2^ ^]^ and nontoxicity.^[^ ^3^ ^]^ HCs are low‐cost materials due to cheap precursors. Often, synthetic organics or biomass are used, e.g., cellulose or lignin, which can be derived from biowaste.^[^ ^4^, ^5^, ^6^ ^]^ In comparison to graphite, HCs show a highly disordered structure.^[^ ^7^, ^8^ ^]^ The macroscopic structure of HCs can be described by discrete fragments of graphenic sheets that are nonplanar, bent, buckled, twisted, and/or rumpled. Due to van der Waals forces, the graphenic sheets cannot be unfolded or flattened and are locally stacked in a highly turbostratic structure.^[^ ^7^, ^8^ ^]^ At a larger scale, HCs contain graphene layers with rather random orientation and voids^[^ ^9^, ^10^ ^]^ covering a wide range of sizes and forms. These voids can have diameters up to 25 nm, and the surface area of HCs ranges from less than 10 to over 100 m^2^ g^−1^.^[^ ^11^, ^12^, ^13^, ^14^, ^15^, ^16^, ^17^, ^18^ ^]^ The resulting structure is often illustrated by the “house of cards” model first proposed by Stevens and Dahn.^[^ ^9^ ^]^

The sodiation of HC shows a typical potential–capacity profile with two distinct regions. The region with a potential above 0.1 V against Na^+^/Na is called the sloping region and the region below 0.1 V plateau region.^[^ ^19^, ^20^ ^]^ Despite extensive research, the detailed storage mechanisms are still under debate. Mainly, three possible active sites for Na^+^ ions are discussed. These are defects (e.g., vacancies, impurities, and dislocations), interlayer spaces, and nanopores. Based on these sites, four different storage models for HC are reported in the literature: the “intercalation filling,” “adsorption intercalation,” “adsorption filling,” and “adsorption intercalation filling” (also known as the “three‐stage model”).^[^ ^3^ ^]^ In these descriptions, the first term describes what happens in the sloping region, while the second and third terms (if they exist) describe the mechanism(s) associated with the plateau region.

Sodium storage at the different active sites in HC leads to different volume changes and therefore different stresses in the electrode. It has been reported in the literature that changes in the volume occur due to the increase of interlayer spacing during sodiation.^[^ ^21^, ^22^ ^]^ Measurements of volume changes by dilatometry^[^ ^23^ ^]^ or mechanical stress by substrate curvature^[^ ^24^ ^]^ were already performed to gain insight into the storage mechanisms. Both reports describe their data using the “intercalation‐filling” model, where sodium intercalation takes place in the sloping region and filling of the nanopores occurs in the plateau region. Here, we further investigate the Na^+^‐ion storage in HC electrodes by the *operando* substrate curvature technique to explore the reaction pathway. In contrast to previous studies,^[^ ^23^, ^24^ ^]^ we investigate the rate dependence of HC and focus on the reversibility of sodium insertion and extraction over several cycles.

## 2 Experimental Section

Borosilicate glass cantilevers with a thickness of 155 μm and an area of 14 mm × 5 mm were used as substrates for the curvature measurements. They were coated by 15 nm of tungsten that serves as an adhesive layer and an additional 25 nm of copper that serves as the current collector. After deposition of the metal films, the cantilevers were spray coated with a HC composite material. For all coated cantilevers, except for the ones used in the galvanostatic intermittent titration technique (GITT) measurement, the following composition was used: 93 wt% HC (Kuranode type II 9 μm, Kuraray), 1.4 wt% conductive carbon (Vulcan XC72R), 1.87 wt% CMC, and 3.73 wt% SBR. For the GITT experiment, a composition of 85 wt% HC (Kuranode type II 5 μm, Kuraray), 5 wt% CMC, and 10 wt% conductive carbon (Vulcan XC72R) was used. The second composition showed stronger changes in stress‐thickness and allowed easier tracking of stress‐thickness relaxations. Although the height of the stress response differs between both cantilevers due to their different composition and density, the general trends that we observe are the same. The coating of the cantilevers was performed using a spray gun. A detailed description of the spray‐coating procedure can be found in Palanisamy et al.^[^ ^25^ ^]^ The cantilevers were covered by a masking tape to provide a 4 mm × 5 mm HC‐free area for the electrical contacts. After spray coating, the cantilevers were dried overnight at room temperature and afterward in a vacuum at 90 °C for 16 h. The cantilevers were stored inside an argon‐filled glove box (O_2_, H_2_O < 0.3 ppm) until usage. Details on mass loading and spray‐coated areas of all cantilevers used in this work can be found in Table S1, Supporting Information.

**Figure 1:**
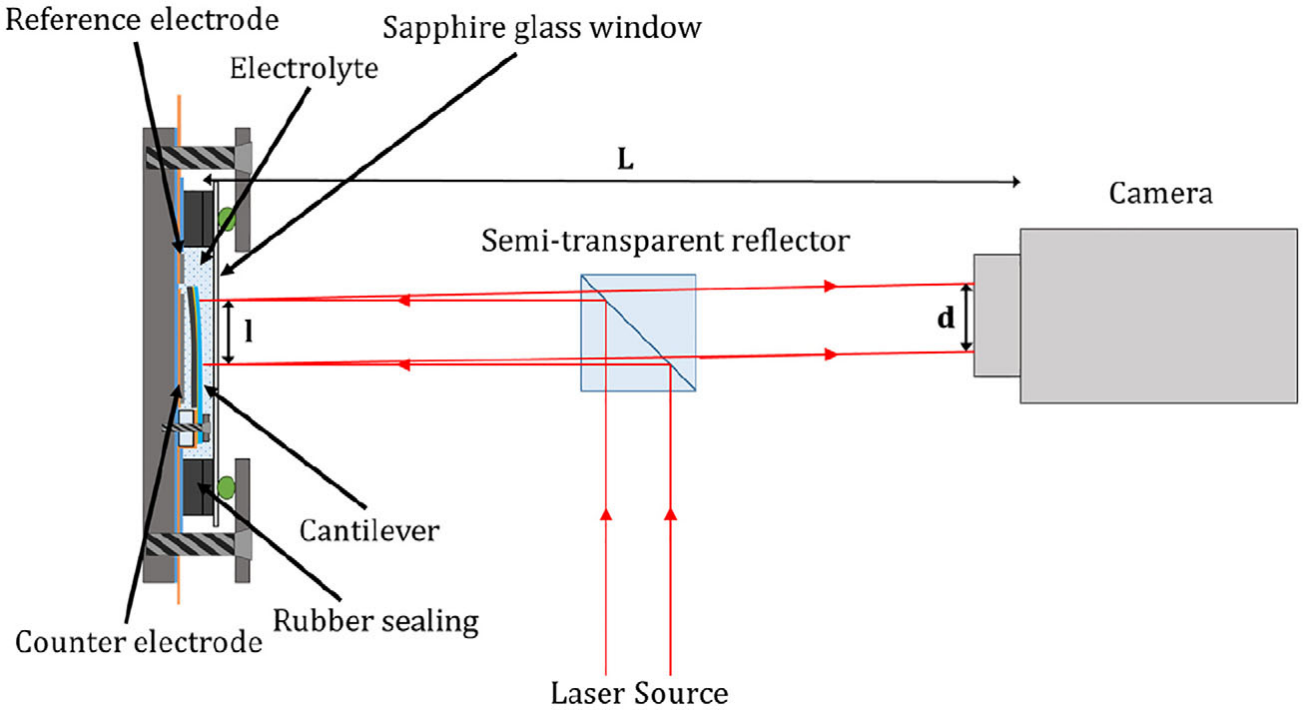
Schematic of the substrate curvature experiment adapted from another study.^[^ ^26^ ^]^

The experimental setup which was used for the curvature measurements was developed by our group in the past^[^ ^26^ ^]^ and is depicted in **Figure** **1**. The setup was optimized for laser‐based measurements through the optical window of a custom‐made electrochemical test cell (Figure S1, Supporting Information). As illustrated in Figure 1, the cantilever was clamped within the test cell and functioned as the working electrode. A three‐electrode configuration was employed to enhance the accuracy of potential measurements. Na metal (99.95% Alfa Aesar) was used as both counter and reference electrode. After assembly, the electrochemical cell was filled with about 0.6 ml electrolyte containing ethylene carbonate (EC) and propylene carbonate (PC), in a 1:1 volume ratio and 1 M NaPF_6_ salt. The water content of less than 3 ppm (determined via the Karl Fischer Titration) was achieved by drying the electrolytes for at least one week with 4 Å molecular sieve (Sigma‐Aldrich). The cell was hermetically sealed by a sapphire window as depicted in Figure 1. After assembly, the cell was transferred from the argon‐filled glove box to the curvature setup. To measure the stress‐thickness, two laser beams (Schäfter + Kirchhoff 51nanoFCM) entered and left the test cell through the transparent sapphire window. The laser beams were reflected at the back of the cantilever, and their position (deflection angle) was recorded by a CMOS camera (Pixelink PL‐B782F) located at a distance of 170–210 mm from the cantilever located inside the cell. Two laser beams were used to exclude the region of the clamp with its irregular stress field from the collected data. More details of the substrate curvature measurements on battery materials can be found in another study.^[^ ^27^ ^]^ Applying straightforward trigonometric calculations, the deflection angles and radius of curvature for the region between the two laser spots were determined. The electrochemical experiments were performed using a commercial battery cycler (VMP3, Bio‐Logic SAS). The camera data (40 Hz) and the electrochemical data from the VMP3 were recorded with a LabVIEW program. To suppress curvature changes due to temperature fluctuations caused by the different thermal expansion coefficients of the composite electrode materials (polymer, metal, and ceramic), the setup was located inside a thermally isolated box. A Peltier‐based active temperature control was used to keep temperature fluctuations below ± 10 mK. All experiments were performed at a constant temperature of 20 °C.

In the early 20th century, Stoney developed the following relation.^[^ ^28^ ^]^

(1) $$\sigma_{f}h_{f}\approx \frac{E_{s}h_{s}^{2}}{6(1-\nu_{s})}*\frac{1}{R}$$

For a film that is much thinner than the substrate $\left(h_{f}\ll \;h_{s}\right)$, the stress‐thickness product $\sigma_{f}\cdot h_{f}$ is proportional to the force at the interface between electrode and substrate and linearly correlates with the inverse radius 1/*R* of the film–substrate bilayer. In this case, the mechanical properties of the investigated film are not required for the calculation of the stress‐thickness, i.e., with $E_{s},\;\nu_{s}$ only the elastic mechanical parameters of the ceramic substrate are needed.

Here, films that have about one‐tenth of the thickness of the underlying substrate were used, and the assumption of a thin film is not fully valid. Moreover, for a porous composite electrode, the source of mechanical stress is the volume expansion of the active material. The active material is mechanically interlinked by a compliant elastic binder that mitigates the stresses of the volume expansions and leads to a reduced stress at the current collector and the underlying substrate. Therefore, the stress‐thickness product is expected to correlate with the volume expansion and shrinkage of the active material in an approximately linear manner—for example, see the roughly linear behavior of a two‐phase reaction with volume expansion during lithium insertion.^[^ ^29^ ^]^ This correlation between stress‐thickness and volume of the active material strongly depends on the amount of binder in the composite electrode and its elastic modulus.^[^ ^30^ ^]^ In this work, only relative volume changes were investigated, and the stress‐thickness product was reported since it can be assumed to be proportional to the volume change of the HC. No attempt was made to quantify volume expansions or report accurate stress levels in the HC.

**Figure 2:**
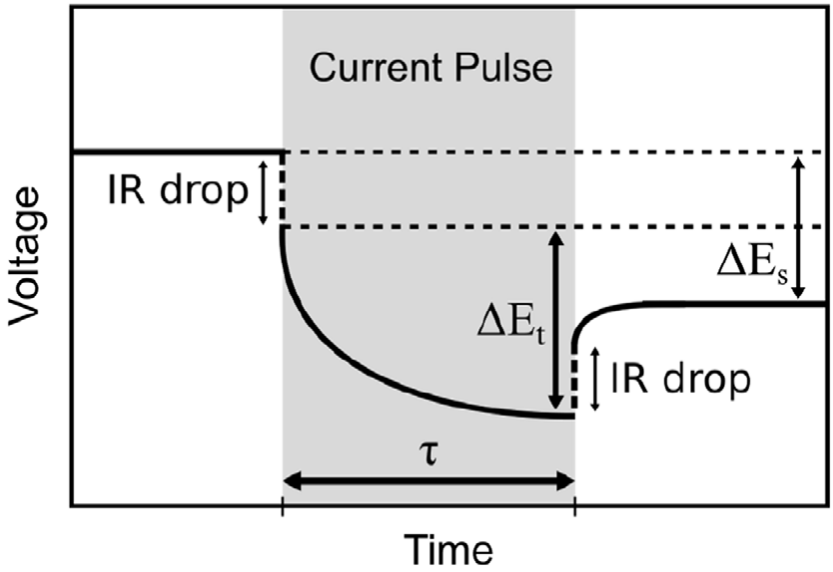
Schematic of the GITT technique derived from another study.^[^ ^12^ ^]^

For the electrochemical cycling, a voltage window of 5 mV to 2 V and a current of 10 mA g^−1^ were chosen. In addition, the GITT was applied in parallel with the *operando* substrate curvature investigations after the first five cycles. The same current of 10 mA g^−1^ as used in the galvanostatic experiment was chosen for all current pulses of the GITT measurements, and the duration of a pulse was 1 h, which was followed by a 30 min open‐circuit potential (OCV) step. In single‐phase regions, when no changes in the storage mechanism were present, diffusion coefficients can be calculated based on Fick's law according to Equation (2).^[^ ^31^ ^]^ In two‐phase regions, it can be problematic to interpret GITT data with respect to diffusion kinetics. Nevertheless, this is often performed, and the results are then termed “apparent diffusivities.” In Equation (2), *τ* is the current pulse time, and *m*_AM_, *V*_m_, and *M*_AM_ are related to the active material and are its mass, molar volume, and molar mass. *S* is the area of the contact surface of the electrode. $\Delta E_{s}$ and $\Delta E_{t}$ are the voltage difference during the OCV phase and the current pulse excluding the IR drop contribution (**Figure** **2**). All other parameters were either constant parameters from the experimental setup or the electrode under investigation. Because in our case *S* is not known and for this work only relative changes of the diffusivity are of interest, only 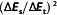 values are reported in this work.

(2) $${\tilde{D}}_{\mathrm{GITT}}=\frac{4}{\pi \;\tau }{\left(\frac{m_{AM}V_{m}}{M_{AM}S}\right)}^{2}{\left(\frac{\Delta E_{s}}{\Delta E_{t}}\right)}^{2},\;\text{with}\;\tau \ll \;\frac{L^{2}}{\tilde{D}}$$

## 3 Results

**Figure 3:**
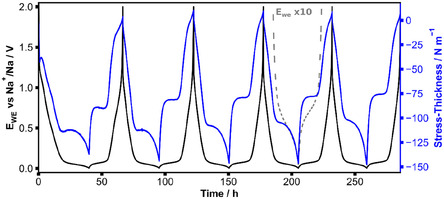
Stress‐thickness data of the first five galvanostatic cycles of a HC composite electrode. A current of 10 mA g^−1^ was applied with a voltage window of 5 mV–2 V versus Na^+^/Na. The dashed gray line shows the electrochemical potential stretched by a factor of 10.

When operated in a typical potential range of 2 V to 5 mV versus Na^+^/Na, the electrochemical potential of HC shows two distinct regions (sloping > 0.1 V and plateau region < 0.1 V), which can be seen in the black curve recorded with a current of 10 mA g^−1^ (**Figure** **3**). Shown in blue are the recorded stress‐thickness values of the *operando* substrate curvature measurements during the galvanostatic cycling. An evolution toward negative stress‐thickness values represents increasing compressive stress, and a motion in the direction of positive values corresponds to increasing tensile stress. The stress‐thickness values were defined to be zero at the beginning of cycling. The data correlates well with the two regimes observed in the electrochemical potential, showing a large change in compressive stress during the sloping region and a much smaller change during the plateau region. At the end of the sodiation, a stronger increase of compressive stress can be observed. The stress‐thickness values corresponding to the same state of charge during sodiation and desodiation are not the same but show a pronounced hysteresis. Substrate curvature experiments on composite electrodes typically contain drift that we attribute to changes in the electrode structure and/or binder motion. This is not the case here, and the initial stress‐thickness of 0 N m^−1^ is almost completely recovered after each cycle. For a detailed comparison of the first five cycles, the potential and stress‐thickness curves are plotted against capacity for cycles one, two, and five in Figure S2, Supporting Information. The first cycle shows a large irreversible capacity of 112 mAh g^−1^ as determined by the comparison of half cycles one and three. Despite this large irreversible capacity, there are only minor differences in the stress levels between these half cycles. The largest difference occurs within the sloping region of the first half cycle, where the slope is reduced in both voltage and stress‐thickness. Besides the difference in the sloping region, half cycles one and three are comparable. There is a small dip directly at the beginning at 8 mAh g^−1^ (0.8 h). The stress‐thickness plateau during sodiation is less pronounced in later cycles when compared to the first and second cycles.

### 3.1 Variation of the Level of Sodiation

**Figure 4:**
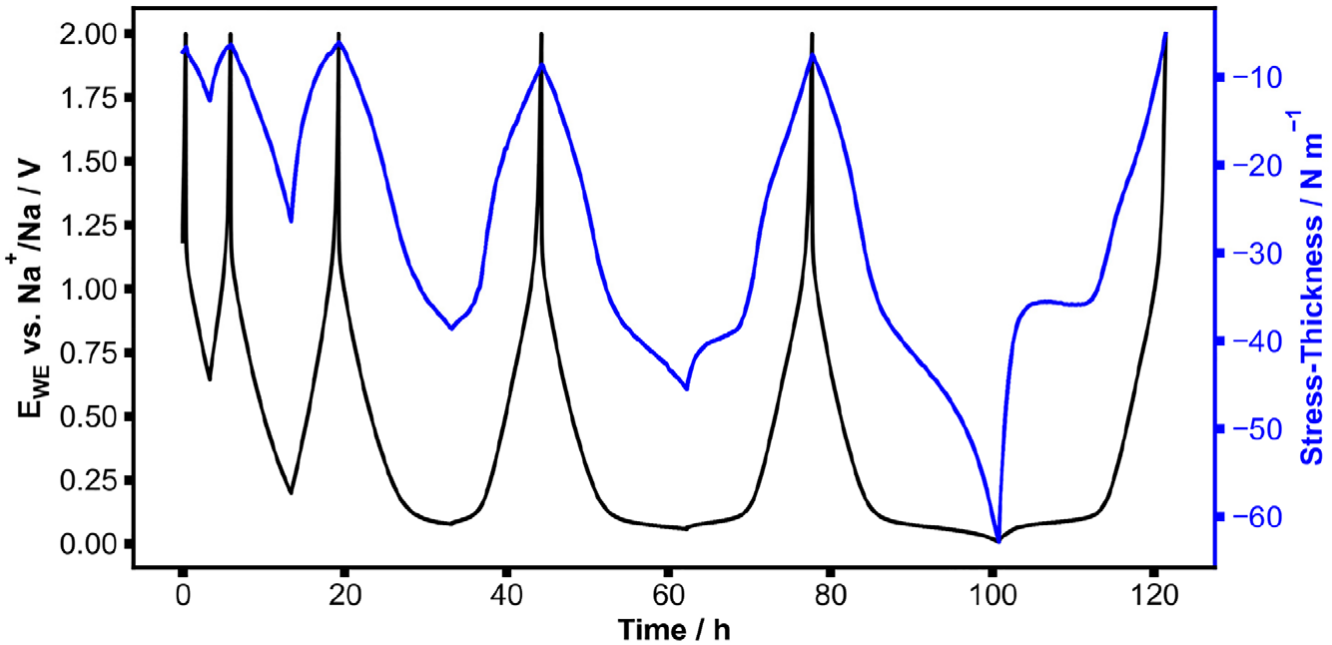
Stress‐thickness data and electrochemical potential of the partial galvanostatically cycled HC composite. A current of 10 mA g^−1^ and the upper potential of 2 V are used. With increasing cycle number, the current is reversed after a capacity of 70 mAh g^−1^, 100 mAh g^−1^, 140 mAh g^−1^, or 180 mAh g^−1^ is reached. The last cycle is a full cycle to 5 mV.

To investigate the mechanical response of the sloping and the plateau region in more detail, partial galvanostatic cycling of a HC cantilever was performed by consecutively increasing the charge corresponding to different levels of sodiation, as shown in **Figure** **4**. While the voltage data is rather reversible between sodiation and desodiation within the five cycles, the mechanical stress data changes, and features emerge in cycles four and five that are strongly asymmetric. This effect correlates with the plateau region of the HC: the stress‐thickness value of the plateau that is present during desodiation is affected by the degree of sodiation in the previous half cycle. For example, after a sodiation of 180 mAh g^−1^ (cycle 4), the stress‐thickness of the desodiation plateau is around −40 N m^−1^, and for full sodiation (cycle 5), the very pronounced desodiation plateau is higher at −36 N m^−1^. The hysteresis in the stress‐thickness curve increases with the level of sodiation.

### 3.2 Effect of Rate

**Figure 5:**
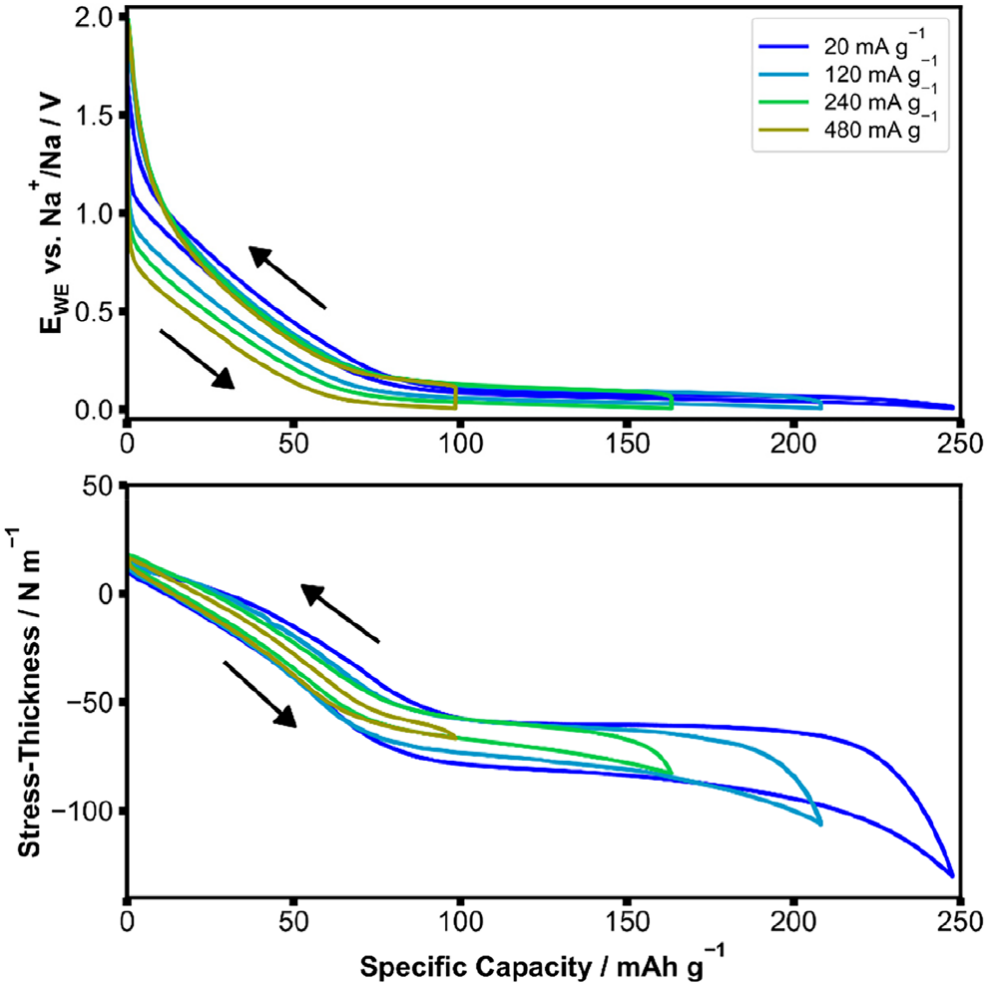
a) Potential and b) stress‐thickness values versus capacity for different rates from 20 to 480 mA g^−1^ of a composite HC spray coated on a cantilever.

Cycling at higher rates leads to smaller capacity and stress‐thickness range (the difference between minimum and maximum within a cycle) as depicted in **Figure** **5**, where the rate was varied between 20 and 480 mA g^−1^, respectively. The observed reduced capacity at higher rates is due to a shortening of the plateau region. In general, increased rates show an increase in the hysteresis in the electrochemical potential and a decrease in the hysteresis in stress‐thickness.

### 3.3 Galvanostatic Titration

**Figure 6:**
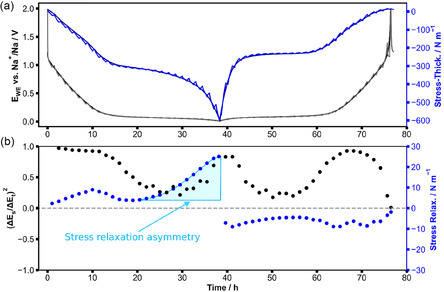
a) GITT of a cantilever spray‐coated with HC composite. After the GITT experiment with its interrupts, a continuous experiment is performed for 51 h. It was performed at the same rate as the galvanostatic periods of the GITT experiment. It is stretched by a factor of 3/2 and overlaid on the GITT data for clarity. The cutoff potentials are 10 mV and 2 V versus Na^+^/Na. The length of the current pulses is 60 min, and the OCV phases are 30 min. b) Blue dots show the difference in stress‐thickness between the start and end of the OCV phases. The mechanical relaxation data show a strong asymmetry between sodiation and desodiation, which is highlighted with the bright blue area. The black dots represent changes in the potential. They were calculated from the voltage changes during OCV according to Equation (2).

To address the dynamics of the electrode, we performed GITT measurements in our substrate curvature experiment on HC (**Figure** **6**). The blue and grey curves are the stress‐thickness and electrochemical potential data of the GITT experiment, which was performed during stable cycling. Immediately after the GITT, a constant current experiment was carried out without the interrupts during GITT. It was performed at the same cycling rate which was used for the galvanostatic periods of the GITT experiment. In Figure 6a, this curve is stretched in time by a factor of 3/2 and overlaid on the GITT data to serve as a reference. The good agreement between both curves indicates that the interrupts do not affect the progression of sodiation and desodiation. The black dots represent 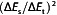, which is proportional to the apparent diffusivity (Equation (2)). The values are determined from the measured potential changes during the current pulse ($\Delta E_{s}$) and the OCV phase ($\Delta E_{t}$). During sodiation, the apparent diffusivity is relatively constant within the sloping region, followed by a strong decrease in the early plateau region with little change until 32 h, where the diffusivity increases again. During desodiation and directly after reversing the current, the diffusivity values remain high for a short period of time. During further desodiation, the diffusivity decreases, staying at a minimum, and then, around 55 h, it rises again. The mechanical relaxation data again show a strong asymmetry between sodiation and desodiation, which is shown by the blue dots and highlighted with the bright blue area in Figure 6b. The relaxation in stress‐thickness changes its sign after the current is reversed. This is not the case for the apparent diffusivity. Even when absolute values are considered, the data of the stress relaxations show less symmetry between sodiation and desodiation than the apparent diffusivity. A significant continuous increase in stress relaxation occurs at the end of the sodiation (starting at 28 h) until the current is reversed for desodiation. During desodiation, the relaxations in stress‐thickness remain at relatively small values below 10 N m^−1^. It seems that the onset of the increase in stress relaxation and diffusivity in the plateau region correlates.

## 4 Discussion

Substrate curvature measurements can provide complementary information to the electrochemistry.^[^ ^24^, ^26^, ^27^ ^]^ Especially for HC electrodes, a large fraction of the reaction takes place at low potentials and only small changes of the electrode potential occur. Here, we additionally measure the mechanical stress‐thickness of the electrode to investigate the reaction of the composite HC electrode with sodium. For porous composite electrodes with soft binders as used here, stress‐thickness can be considered to be proportional to the change in volume of the active material. The data in Figure 3 shows the high sensitivity of the substrate curvature method. The mechanical data originates from the electrochemical processes and contains many features. For example, in Figure 3, the plateaus in the stress‐thickness are clear, and their surroundings are well resolved. The *operando* cells are stable, and the HC electrodes are sufficiently reliable, as shown from the reproducibility of the data between samples (Figure S4, S5, and S7, Supporting Information) and from cycle to cycle (Figure 3). For each sample and cycle, the stress‐thickness level of the sodiation and desodiation plateau and the stress‐thickness range are shown in Figure S7, Supporting Information. In this work, we compare electrochemistry and volume changes to infer reactions of HC with sodium. First, we focus on the sodium loss in the first cycle, and second, we investigate the reaction pathway at later cycles with a particular focus on its reversibility.

### 4.1 Sodium Loss during First Cycle

During the first sodiation, HC electrodes always experience losses in capacity. According to the literature, these losses are related to the structural features of the HC and the electrochemical conditions. Values for the initial Coulombic efficiency range between 73.6% and 95%.^[^ ^19^ ^]^ Our spray‐coated composite HC cantilever‐based electrodes show initial Coulombic efficiencies that are lower than the values reported in the literature, with values between 67% and 74% (Figure S2, Supporting Information). In our test cell, the spray‐coated electrode with its high surface to volume ratio is immersed within large amounts of electrolyte. This may lead to reduced Coulombic efficiencies. To date, the loss of sodium in the first cycle is not yet fully understood, and different underlying mechanisms are discussed in the literature.^[^ ^19^ ^]^ Reactions that are often considered are 1) the growth of SEI, 2) trapping of Na in the active material, and 3) possible side reactions with components of the electrode such as binder or conductive carbon.^[^ ^19^ ^]^ We expect that these side reactions all affect the volume change of the first sodiation cycle in different ways. The data in the literature is not coherent: electrochemical dilatometry of HC carbonized at different temperatures reported in the supplemental data by Alptekin et al.^[^ ^21^ ^]^ shows an irreversible increase in volume in the first cycle of HC that strongly depends on the structure of the HC. A close inspection of the data presented in Escher et al.^[^ ^23^ ^]^ shows that even a shrinkage can occur in the first full cycle.

Despite the large differences in the capacity of the first and the second sodiation cycles, the stress‐thickness values after both cycles are very similar in Figure 3. This means that the irreversibly stored sodium does not lead to a change in the volume of the electrode. Based on the measured capacities in the sloping and plateau regions, it can be concluded that most of the sodium losses occur in the sloping region of the first cycle (Figure S3, Supporting Information). The plateau region contributes less to the irreversibility.

The stress‐thickness in Figure 3, S2, Supporting Information, shows a strong drop within the first ≈50 min (8 mAh g^−1^) of sodiation leading to a minimum and a subsequent slower rise of compressive stress. We interpret the initial evolution of stress by a strong expansion of the structurally more or less undistorted HC caused by the insertion of relatively small amounts of sodium. The minimum may be caused by the onset of larger structural changes of the electrode. After overcoming the minimum, the stress data is linear again between ≈1 V and ≈0.1 V (−40 N m^−1^ and −115 N m^−1^) but exhibits a reduced slope (Figure S2b, Supporting Information) compared to the second insertion. This smaller volume increase per charge is probably caused by larger structural changes of the electrode. During the first sodiation, the capacity between 1 V and 0.1 V is increased compared to the second sodiation, while the changes in volume (stress‐thickness) are similar. This implies that sodium is irreversibly stored in the HC matrix or in defects where it does not contribute to the volume expansion of the active material. With our method, we cannot separate and quantify the different components of Na loss (i, ii, and iii above). Side reactions with the binder or carbon black (iii) cannot be fully excluded, but the large irreversible capacities observed in our experiments in combination with the limited contributions of these components (5.6 wt% and 1.4 wt%) reveal that these side reactions are probably not significant. Moreover, irreversible SEI formation 1) would lead to a volume expansion and cannot cause a contraction and therefore is not compatible with the reduced volume expansion per charge that is measured during first sodiation. Figure S6, Supporting Information, shows data from a cell with a different electrolyte that is expected to lead to a different SEI. The characteristics of the stress‐thickness data are not altered. In contrast to the side reactions 1) and 3), a structural change of the active materials during sodiation 2) appears to be likely based on the observed characteristic shape of the stress‐thickness data: first, there is a high slope of the stress‐thickness, i.e., a large volume change per sodium atom that may be due to a solid solution or topotactic insertion of sodium. Second, a minimum in the stress‐thickness forms, which may be caused by the nucleation of a sodium‐rich phase where excess compressive stresses are needed to overcome the yield stress of one of the two interacting phases. Third, a linear increase in stress with a lower slope is probably caused by a phase coexistence where the sodium‐rich phase gradually grows at the expense of the initial HC.

### 4.2 Asymmetric Sodiation and Desodiation

The storage mechanism of sodium in HC has not yet been fully clarified. In the rest of this discussion section, we are going to combine our mechanical and electrochemical data to investigate the reaction pathway during operation of the cell beyond the initial cycle. The general trends in our data can also be found in electrochemical dilatometry data where electrode thickness was directly recorded.^[^ ^21^ ^]^ The investigations based on substrate curvature measurements^[^ ^24^ ^]^ and dilatometry^[^ ^23^ ^]^ suggest the intercalation of Na^+^ ions in the sloping region and pore filling in the plateau region. Also, *operando* Raman spectroscopy measurements support this sequence.^[^ ^16^, ^32^ ^]^ These and other studies are in line with the intercalation‐filling model proposed by Stevens and Dahn.^[^ ^33^ ^]^ Pore filling on the plateau region is also confirmed by NMR measurements.^[^ ^34^ ^]^ All of these *operando* measurements either suggest or cannot rule out the possibility of Na adsorption at defect sites during the sloping region, which was suggested by Alptekin et al.^[^ ^35^ ^]^ Our stress‐thickness data correlates with volume changes, and we expect very little sensitivity to surface effects such as the adsorption of Na^+^‐ions.

At first glance, with its sequence of sloping regions and plateaus, our stress‐thickness data are in line with sequential sodium insertion and pore filling. The steep slopes in the stress‐thickness data agree with the insertion/extraction, and the relatively flat plateaus in the mechanical stress indicate that the filling and the depletion of pores do not cause significant volume changes of the HC. This is expected for empty pores that are located inside the active material. New aspects with respect to the reaction pathways become apparent by the comparison of sodiation and desodiation: the mechanical data reveals that sodium insertion and extraction are not symmetric with respect to charge, e.g., the data shown in Figure 3 is not mirror symmetric around 150 h, where the current is reversed. Here, we use the term symmetry to describe the mirror symmetry with respect to charge, which is equivalent to a forward‐backward symmetry of the reaction pathway between sodiation and desodiation. The observed asymmetry between sodiation and desodiation is present in the voltage but is more evident in the stress‐thickness data. For example, it can be seen that the stress‐thickness levels of the plateau differ during sodiation and desodiation by roughly 25 N m^−1^, which is more than 15% of the total stress‐thickness (volume) change of HC. This large mechanical effect can be hardly traced back to the pore‐filling mechanism with its small effects on the stress‐thickness.

GITT measurements, reported in the literature, indicate that the storage mechanism in the plateau region is more complex than suggested by the simple insertion/filling model. Bommier et al.^[^ ^36^ ^]^ observed a change in apparent diffusivity at the end of the plateau region, similar to what is observed in the voltage relaxations in Figure 6b. They related this behavior to a change in the storage mechanism and introduced the three‐stage model. Sodiation starts with adsorption in the sloping region and transitions to the plateau, where intercalation and later pore filling take place.^[^ ^36^ ^]^ Alptekin et al.^[^ ^21^ ^]^ confirmed this three‐stage model by experimental and computational results of HCs prepared by hydrothermal carbonization at different temperatures. In contrast to Bommier et al.,^[^ ^36^ ^]^ they assigned the intercalation to the sloping region, which is also in agreement with our data in which larger volume changes happen in the sloping region. Increasing the carbonization temperature led to different HC structures with lower capacities in the sloping region but higher plateau capacities.^[^ ^21^ ^]^ This was explained by a decreased number of defects and a smaller interlayer spacing, reducing the sodium storage in the sloping region, and an increase in closed porosity, increasing pore filling and plateau capacity. At the highest carbonization temperatures, the capacities decreased. This was explained by fewer crystal defects and reduced interlayer spacing in the HC, which reduces transport and makes the separated closed pores inaccessible for sodium.^[^ ^21^ ^]^

These observations indicate that the transport of sodium through the HC and the filling of pores are not independent, and in contrast to the simple intercalation‐filling model, both mechanisms do not operate sequentially. Intercalation is not only a storage mechanism, it also requires the transport of guest ions through the host lattice. The closed pores that are encountered in HCs are not accessible from the electrolyte and can only be reached through the bulk of the HC by diffusion. The atomic structure of the HC is highly distorted and contains many lattice defects. The transport of sodium to the pores requires the diffusion of sodium through these defects, e.g., along dislocations or grain boundaries. We hypothesize that the diffusivity depends on the structure of the defects that is interlinked with the amount of sodium contained in them.

The stress‐thickness in Figure 4 shows that the degree of symmetry of the reaction depends on the level of sodiation of the HC. The asymmetry starts when pores are filled and increases with the amount of sodium stored in the pores. Similarly, Figure 5 shows that at high rates, pores are not filled anymore and that the reactions become more symmetric, which can be seen from the hysteresis in Figure 5b. These observations indicate that the postulated interaction between intercalation and pore filling via the altered transport properties of HC can already explain a reaction asymmetry without the need for the introduction of additional mechanisms. In Figure 3, the characteristic shape of the stress‐thickness is not symmetric between sodiation and desodiation. During sodiation, the stress‐thickness changes gradually toward the end of sodiation, while it abruptly changes during desodiation. The abrupt change occupies almost half of the total stress‐thickness change. Not many mechanisms can account for such large volume changes. Based on the current knowledge in this field and our stress‐thickness data, intercalation of sodium is the mechanism that leads to the largest volume changes. Obviously, the filling and depletion of pores cannot account for the observed very large stress changes and the asymmetry. The GITT data in Figure 6 gives further insights: the apparent diffusivity data (black dots in Figure 6b) are not centered around the point of current reversal, an effect that was also shown using GITT measurements in the literature.^[^ ^37^ ^]^ The measured values at the beginning of desodiation between 38 h and 40 h are higher than the ones at the end of sodiation (between 36 h and 38 h). Given these large differences around the same state of charge, the values may not be considered as diffusivity but rather can be seen as indicators for the large differences in the electrochemical reactions around the point of current reversal. For example, a two‐phase reaction toward the end of sodiation or the formation of Na clusters^[^ ^37^ ^]^ could explain deviations from the single‐phase behavior that complicate the analysis as diffusion data. The relaxations in the stress‐thickness data (Figure 6b) are also highly asymmetric, and deviations from the symmetry are apparent toward the end of the sodiation plateau, where the stress‐thickness strongly relaxes toward a smaller electrode volume once the sodiation is interrupted.

**Figure 7:**
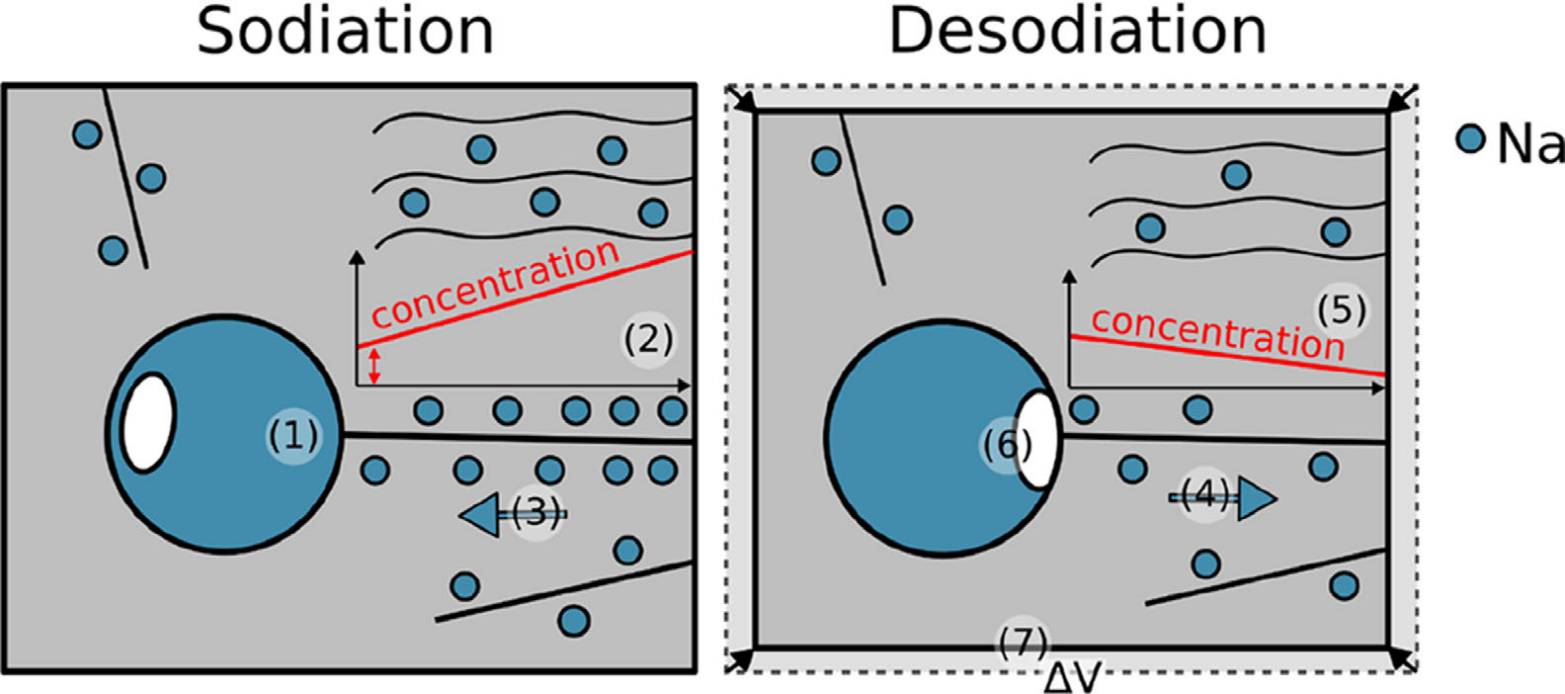
Schematic of modified storage model. The particle exhibits a different volume at the same state of charge due to a different amount of sodium within the intercalation sites.

The schematic in **Figure** **7** summarizes our idea of the interaction of intercalation and pore filling in HC and its consequences on the particle volume. Once pores become increasingly filled, (1) in Figure 7, higher driving forces are required for the insertion of sodium. As a consequence, the potential at the particle surface lowers and the concentration gradient toward the pore increases (2). This drives additional sodium into the already occupied transport pathways and locally increases the volume. As transport pathways, we consider graphitic layers and crystal defects where sodium can intercalate but also move (3). We further assume that higher concentrations of sodium in these paths lead to an expansion of the HC. Desodiation reverses the direction of the sodium ions (4). The sodium concentration at the surface of the particle decreases, and the potential rises so that concentration gradients change their direction to move sodium toward the surface (5). The potential of HC in these highly sodiated states depends very little on the sodium concentration, and therefore small changes on the surface may be hardly detectable in the electrochemical data. Sodiation requires higher average Na^+^ concentrations in the transport paths, leading to volume expansion of the HC, while desodiation releases Na^+^ at lower concentrations, causing contraction. Hence, at the same state of charge, the same particle has different volumes during sodiation and desodiation. If filling and depletion of a pore require different energies, then a different amount of sodium would be present in the paths to and from the pore. Figure 7 depicts an example where the energy difference between pore filling and depletion is a consequence of different geometries of the sodium inside the pore and where a local depletion (6) during dissolution can obstruct the transition between pore and defect. During desodiation, the transport path and other regions further deintercalate, leading to a reduction of the volume of the active particle (7).

During interrupts, concentration gradients relax, and both storage mechanisms interact. The observed volume contraction during sodiation may be explained by the motion of sodium out of the transport path into the pores. This deintercalation results in large volume changes versus time (Figure 6b). The different levels of intercalation of sodium in the defects during sodiation and desodiation may not only exist in the active transport paths. They should also be present in regions that do not feed sodium to pores. The large number of intercalation sites that are available in HC amplify the described effect and could explain the observed large difference in electrode volume between pore filling and depletion.

## 5 Conclusion

*Operando* substrate curvature measurements were used to track volume changes in composite HC electrodes, which were spray coated onto cantilevers and interpreted to elucidate the fundamental storage mechanism of sodium and its reversibility. In the first cycle, sodium is irreversibly lost. This happens mostly in the sloping region, and the trapped sodium does not cause a significant change of the volume of the HC. The mechanical signature is in agreement with the nucleation and subsequent growth of a sodiated phase.

In the cycles following the first sodiation, our data generally agrees with the intercalation‐pore‐filling model. The mechanical data exhibits a high sensitivity and reveals a strong asymmetry between sodiation and desodiation. To date, intercalation and pore filling are considered to be consecutive. We now suggest that both mechanisms interact: intercalation into HC locally changes its structure, and the filling of closed pores requires transport of sodium through the intercalated HC. In this way, the structure of the HC and the associated sodium mobility along the intercalated path to a pore affect its filling. During sodiation and desodiation, very different sodium concentrations exist at the perimeter of a HC particle, where sodium is present in excess during insertion and deficient during desodiation. Our measurements show that pore filling alone has little effect on the volume of particles, while intercalation has a pronounced effect. We therefore interpret the discrepancy in volume between the filling and the depletion of pores with different sodium concentrations in the transport pathways through the HC. Origins of this difference may be an asymmetry between pore filling and depletion and/or a mobility of sodium that depends on its concentration, i.e., a different level of intercalation inside the transport path.

HCs differ in their structure dependent on their precursors and synthesis routes. Although we consider the coupling between intercalation and pore filling to be a general phenomenon, we suspect that HCs with different defect structures and consequently different transport paths vary in their asymmetry. Our results indicate that the structure of the HC plays an important role not only for (de)intercalation but also for the filling and depletion of pores. In the development of HC electrodes, attention should be given not only to the distribution of pore sizes but also to the intercalation path that is a prerequisite for facile pore filling and depletion.

## Conflict of Interest

The authors declare no conflict of interest.

## Supporting information

Supplementary Material

## Data Availability

The data that support the findings of this study are available from the corresponding author upon reasonable request.

## References

[1] H. Moriwake , A. Kuwabara , C. A. Fisher , Y. Ikuhara , RSC Adv. 2017, 7, 36550.

[2] F. Xie , Z. Xu , Z. Guo , Y. Li , Y. Lu , M. M. Titirici , Y. S. Hu , Sodium‐Ion Batteries: Materials, Characterization, and Technology 2022, 1, 27.

[3] F. Xie , Z. Xu , Z. Guo , M.‐M. Titirici , Prog. Energy 2020, 2, 042002.

[4] P. C. Rath , J. Patra , H.‐T. Huang , D. Bresser , T.‐Y. Wu , J.‐K. Chang , ChemSusChem 2019, 12, 2302. 30835938 10.1002/cssc.201900319

[5] J. Wang , P. Nie , B. Ding , S. Dong , X. Hao , H. Dou , X. Zhang , J. Mater. Chem. A 2017, 5, 2411.

[6] L. Liu , F. Xu , A. Zou , Z. Yu , J. Jiang , S. Yin , B. Weng , Mater. Today Commun. 2024, 41, 110271.

[7] P. J. Harris , Crit. Rev. Solid State Mater. Sci. 2005, 30, 235.

[8] Z. Li , C. Bommier , Z. S. Chong , Z. Jian , T. W. Surta , X. Wang , Z. Xing , J. C. Neuefeind , W. F. Stickle , M. Dolgos , P. A. Greaney , X. Ji , Adv. Energy Mater. 2017, 7, 1602894.

[9] D. A. Stevens , J. R. Dahn , J. Electrochem. Soc. 2001, 148, A803.

[10] I. A. S. Edwards , H. Marsh , R. Menendez , B. Rand , S. West , A. J. Hosty , K. Kuo , B. McEnaney , T. Mays , D. J. Johnson , J. W. Patrick , D. E. Clarke , J. C. Crelling , R. J. Gray , Introduction To Carbon Science, 1 ed., Butterworth‐Heinemann, Oxford, Stoneham, MA 1989.

[11] X. Dou , I. Hasa , D. Saurel , C. Vaalma , L. Wu , D. Buchholz , D. Bresser , S. Komaba , S. Passerini , Mater. Today 2019, 23, 87.

[12] W. Luo , C. Bommier , Z. Jian , X. Li , R. Carter , S. Vail , Y. Lu , J.‐J. Lee , X. Ji , ACS Appl. Mater. Interfaces 2015, 7, 2626. 25562593 10.1021/am507679x

[13] C. Bommier , W. Luo , W.‐Y. Gao , A. Greaney , S. Ma , X. Ji , Carbon 2014, 76, 165.

[14] J. Jin , Z.‐Q. Shi , C.‐Y. Wang , Electrochimica Acta 2014, 141, 302.

[15] F. Wu , M. Zhang , Y. Bai , X. Wang , R. Dong , C. Wu , ACS Appl. Mater. Interfaces 2019, 11, 12554. 30875192 10.1021/acsami.9b01419

[16] S. Komaba , W. Murata , T. Ishikawa , N. Yabuuchi , T. Ozeki , T. Nakayama , A. Ogata , K. Gotoh , K. Fujiwara , Adv. Funct. Mater. 2011, 21, 3859.

[17] Z. Hong , Y. Zhen , Y. Ruan , M. Kang , K. Zhou , J. M. Zhang , Z. Huang , M. Wei , Adv. Mater. 2018, 30, 1802035. 10.1002/adma.201802035 29808566

[18] M. Dahbi , M. Kiso , K. Kubota , T. Horiba , T. Chafik , K. Hida , T. Matsuyama , S. Komaba , J. Mater. Chem. A 2017, 5, 9917.

[19] Y. Yang , C. Wu , X. X. He , J. Zhao , Z. Yang , L. Li , X. Wu , L. Li , S. L. Chou , Adv. Funct. Mater. 2024, 34, 2302277.

[20] B. Zhang , C. M. Ghimbeu , C. Laberty , C. Vix‐Guterl , J. M. Tarascon , Adv. Energy Mater. 2016, 6, 1501588.

[21] H. Alptekin , H. Au , A. C. Jensen , E. Olsson , M. Goktas , T. F. Headen , P. Adelhelm , Q. Cai , A. J. Drew , M.‐M. Titirici , ACS Appl. Energy Mater. 2020, 3, 9918.

[22] K. Wang , Y. Xu , Y. Li , V. Dravid , J. Wu , Y. Huang , J. Mater. Chem. A 2019, 7, 3327.

[23] I. Escher , G. A. Ferrero , M. Goktas , P. Adelhelm , Adv. Mater. Interfaces 2022, 9, 2100596.

[24] A. Chanda , A. Pakhare , A. Alfadhli , V. A. Sethuraman , S. P. Nadimpalli , J. Power Sources 2024, 609, 234678.

[25] K. Palanisamy , S. Daboss , D. Schäfer , M. Rohnke , L. Derr , M. Lang , R. Schuster , C. Kranz , Batteries Supercaps 2024, 7, e202300402.

[26] Z. Choi , D. Kramer , R. Mönig , J. Power Sources 2013, 240, 245.

[27] D. Schäfer , K. Hankins , M. Allion , U. Krewer , F. Karcher , L. Derr , R. Schuster , J. Maibach , S. Mück , D. Kramer , R. Mönig , F. Jeschull , S. Daboss , T. Philipp , G. Neusser , J. Romer , K. Palanisamy , C. Kranz , F. Buchner , R. J. Behm , A. Ahmadian , C. Kübel , I. Mohammad , A. Samoson , R. Witter , B. Smarsly , M. Rohnke , Adv. Energy Mater. 2024, 14, 2302830.

[28] G. G. Stoney , Proc. R. Soc. London, Ser. A., Containing Papers of a Mathematical and Physical Character 1909, 82, 172.

[29] M. Janzen , D. Kramer , R. Mönig , Energy Technol. 2021, 9, 2000867.

[30] T. Brendel , M. Janzen , M. Müller , W. Bauer , D. Kramer , R. Mönig , Energy Technol. n/a, 2500106, 10.1002/ente.202500106.

[31] W. Weppner , R. A. Huggins , Js Solid State Chem. 1977, 22, 297.

[32] H. Euchner , B. P. Vinayan , M. A. Reddy , M. Fichtner , A. Groß , J. Mater. Chem. A 2020, 8, 14205.

[33] D. Stevens , J. Dahn , J. Electrochem. Soc. 2000, 147, 1271.

[34] J. M. Stratford , A. K. Kleppe , D. S. Keeble , P. A. Chater , S. S. Meysami , C. J. Wright , J. Barker , M.‐M. Titirici , P. K. Allan , C. P. Grey , J. Am. Chem. Soc. 2021, 143, 14274. 34431677 10.1021/jacs.1c06058

[35] H. Au , H. Alptekin , A. C. Jensen , E. Olsson , C. A. O’Keefe , T. Smith , M. Crespo‐Ribadeneyra , T. F. Headen , C. P. Grey , Q. Cai , A. J. Drew , M.‐M. Titirici , Energy Environ. Sci. 2020, 13, 3469.

[36] C. Bommier , T. W. Surta , M. Dolgos , X. Ji , Nano Lett. 2015, 15, 5888. 26241159 10.1021/acs.nanolett.5b01969

[37] Y. Aniskevich , J. H. Yu , J. Y. Kim , S. Komaba , S. T. Myung , Adv. Energy Mater. 2024, 14, 2304300.

