# Supplementary material for: Mechanical Stress Reveals Asymmetry of Sodiation and Desodiation of Hard Carbon
Source: ChemSusChem. 2025 Aug 19;18(19):e202501272. doi: 10.1002/cssc.202501272 (PMC12487754; doi:10.1002/cssc.202501272)
Supplement: Supplementary file 1 — Supplementary Material [file CSSC-18-e202501272-s001.pdf]

Mechanical Stress Reveals Asymmetry of Sodiation and Desodiation of Hard Carbon

Stefan Mück[a], Dominik Kramer* [a], Krishnaveni Palanisamy[b], Christine Kranz[b], and Reiner Mönig[a]

[a] Karlsruhe Institute of Technology
Institute for Applied Materials
Hermann-von-Helmholtz-Platz 1, 76344 Eggenstein-Leopoldshafen, Germany
E-mail: dominik.kramer@kit.edu

[b] Ulm University
Institute of Analytical and Bioanalytical Chemistry
Albert-Einstein-Allee 11, 89081 Ulm, Germany

Supporting Information

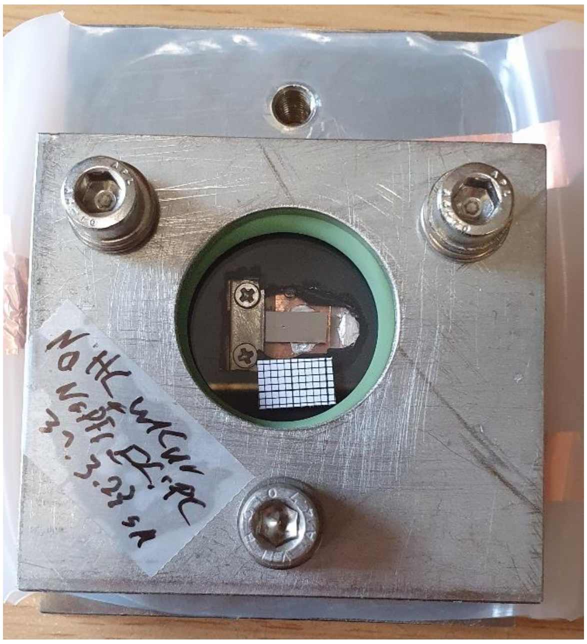

Figure S1. Photograph of the *operando* substrate curvature cell based on stainless steel parts. The cantilever (ca. 15 mm x 4 mm) is rigidly clamped in a small compartment filled with electrolyte behind an optical window. Besides working and counter electrode, a sodium reference electrode is used for measuring the correct electrode potential.

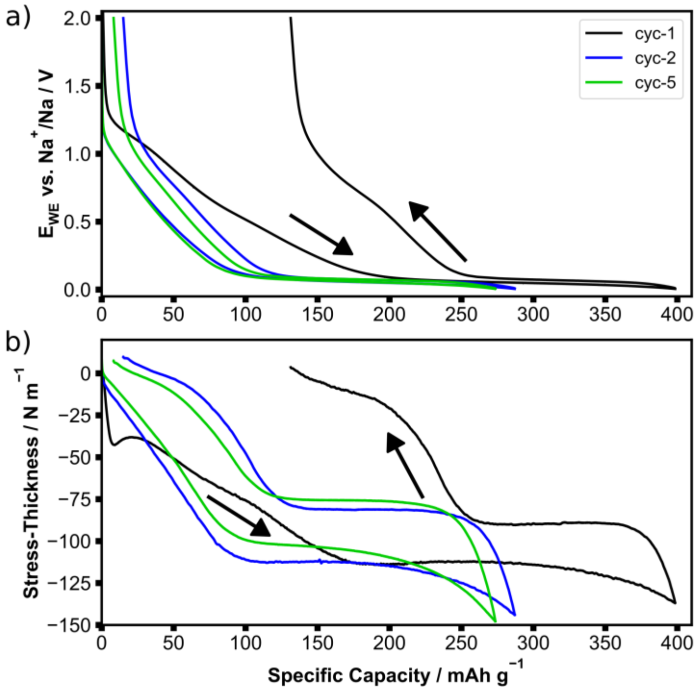

Figure S2. Potential (a) and stress-thickness (b) of the first, second and fifth cycle (10 mA g^{-1}) from Figure 3 plot against capacity.

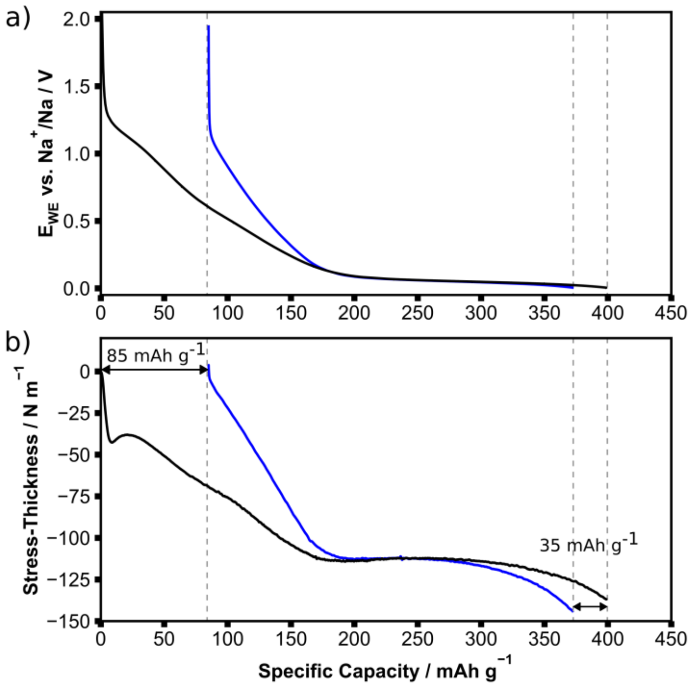

Figure S3. Potential and stress-thickness of the first (black) and second (blue) sodiation cycle. The second sodiation is shifted along the capacity axis so that the plateau and sloping region of both sodiation match and their respective capacity can easily be identified.

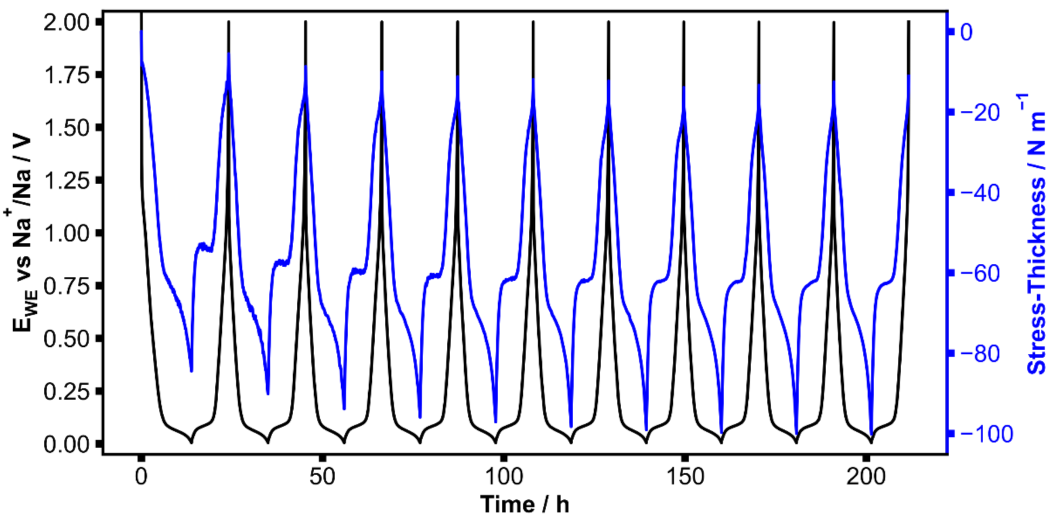

Figure S4. Stress-thickness data of the first ten galvanostatic cycles of a HC composite electrode (different specimen). A current of 25 mA g^{-1} was applied with a voltage window of 5 mV to 2 V vs. Na^+/Na.

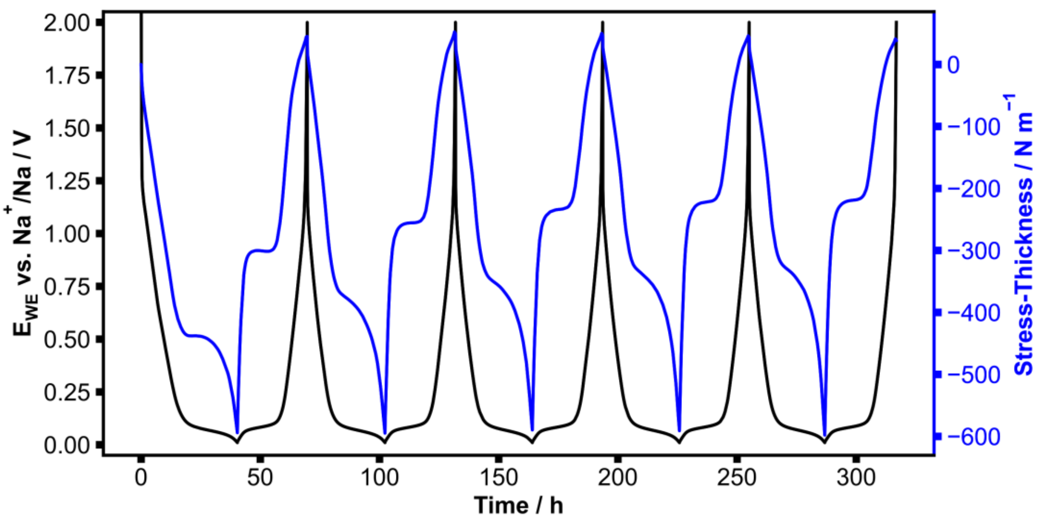

Figure S5. Stress-thickness data of the first five galvanostatic cycles of a HC composite electrode (different specimen, same composition as GITT sample). A current of 10 mA g^{-1} was applied with a voltage window of 5 mV to 2 V vs. Na^+/Na.

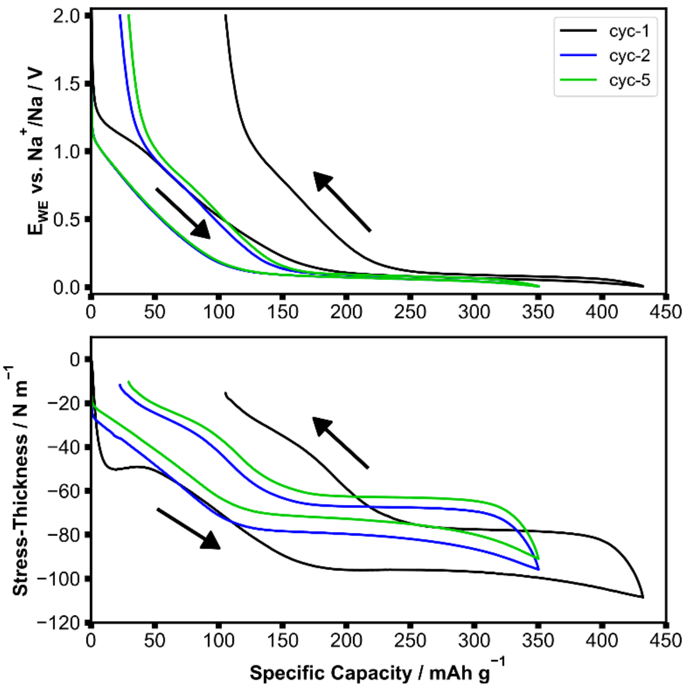

Figure S6. Stress-thickness data of the first, second and fifth galvanostatic cycles of a HC composite electrode (different specimen). For this measurement a different electrolyte was used (1 M $NaPF_6$ dissolved in diglyme). A current of 10 mA g^{-1} was applied with a voltage window of 5 mV to 2 V vs. Na^+/Na.

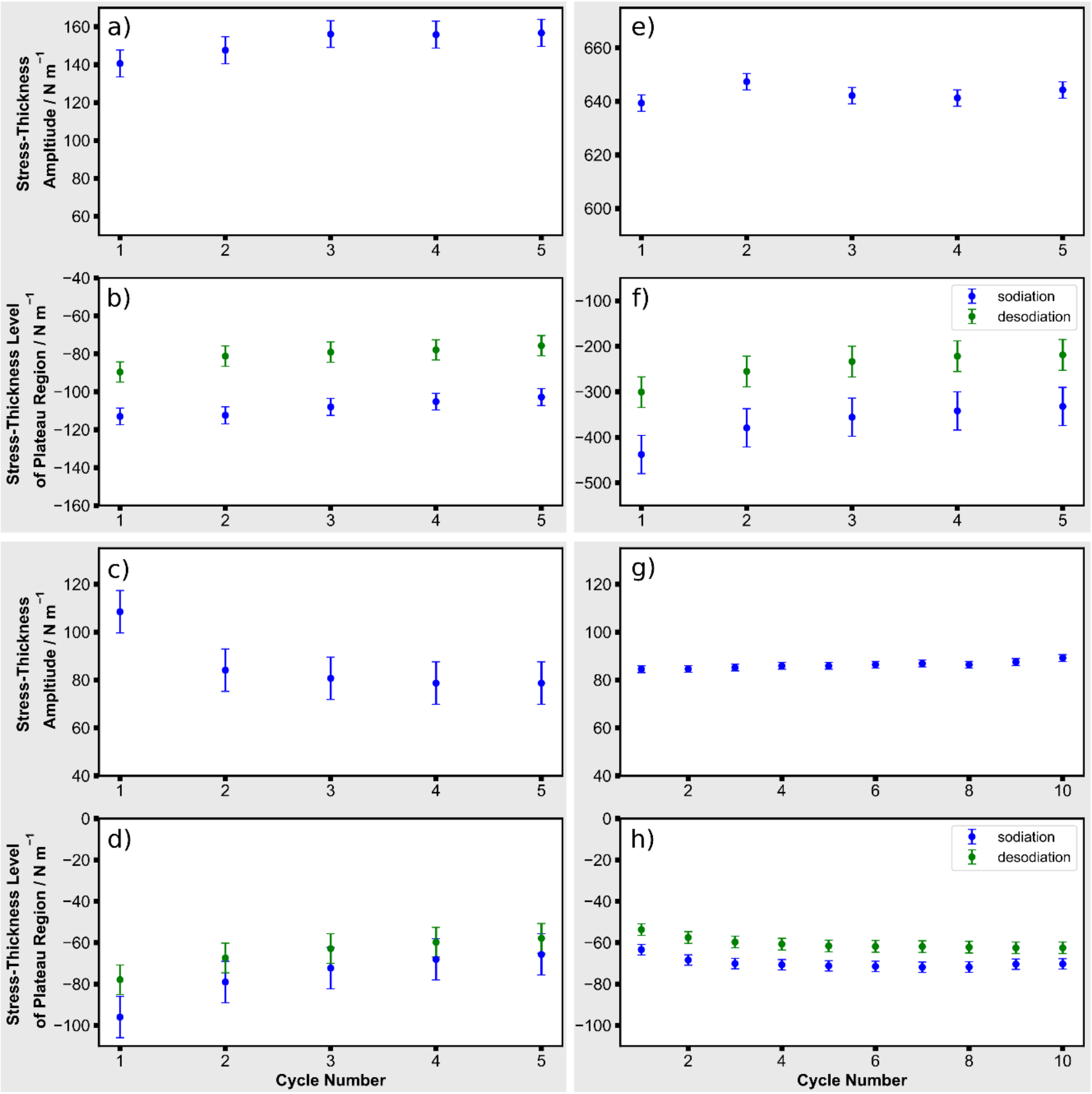

Figure S7. Stress-thickness range of the first cycles from four different samples are shown in a), c), e) and g). The stress levels of the sodiation and desodiation plateaus from the same samples and cycles are shown in b), d), f) and h). a) and b) belong to the same sample as Figure 3 and S2. c) and d) belong to the sample from Figure S6. e) and f) belong to the sample shown in Figure S5. g) and h) belong to the sample in Figure S4. The error bars represent the standard deviation of the respective value within one sample/cell.

Figure S7 shows ranges and the plateau levels of the stress-thickness of four different samples. Besides S7 e) and f) the electrodes were produced in the same deposition run by spray coating of HC with CMC/SBR binder. Our thin spray coating was not uniform over larger distances and the tested electrodes/cantilevers were taken from different regions. Probably variations in coating thickness explain the characteristic differences in the stress-thickness values of the three samples. The cantilever in Figure S7 e) and f) only contains CMC binder and therefore shows higher stresses.

Sample depicted in Figure S7	Spray coated area in cm²	Mass in mg	Mass loading in mg/cm²
a), b)	0.41	0.56	1.37
c), d)	0.46	0.48	1.04
e), f)	0.39	0.87	2.23
g), h)	0.38	0.32	0.84

Table S1. Area and mass loading of investigated cantilevers
